# Engineering Mycobacteria for the Production of Self-Assembling Biopolyesters Displaying Mycobacterial Antigens for Use as a Tuberculosis Vaccine

**DOI:** 10.1128/AEM.02289-16

**Published:** 2017-02-15

**Authors:** Jason W. Lee, Natalie A. Parlane, Bernd H. A. Rehm, Bryce M. Buddle, Axel Heiser

**Affiliations:** a Institute of Fundamental Sciences, Massey University, Palmerston North, New Zealand; b AgResearch, Hopkirk Research Institute, Palmerston North, New Zealand; c PolyBatics, Palmerston North, New Zealand; University of Toronto

**Keywords:** Mycobacterium smegmatis, polyhydroxyalkanoate synthesis, tuberculosis, tuberculosis vaccines

## Abstract

Tuberculosis (TB) is a disease caused by Mycobacterium tuberculosis or Mycobacterium bovis and still remains one of the world's biggest global health burdens. Recently, engineered polyhydroxyalkanoate (PHA) biobeads that were produced in both Escherichia coli and Lactococcus lactis and displayed mycobacterial antigens were found to induce significant cell-mediated immune responses in mice. We observed that such PHA beads contained host cell proteins as impurities, which we hypothesized to have the potential to induce immunity. In this study, we aimed to develop PHA beads produced in mycobacteria (mycobacterial PHA biobeads [MBB]) and test their potential as a TB vaccine in a mouse model. As a model organism, nonpathogenic Mycobacterium smegmatis was engineered to produce MBB or MBB with immobilized mycobacterial antigens Ag85A and ESAT-6 on their surface (A:E-MBB). Three key enzymes involved in the poly(3-hydroxybutyric acid) pathway, namely, β-ketothiolase (PhaA), acetoacetyl-coenzyme A reductase (PhaB), and PHA synthase (PhaC), were engineered into E. coli-Mycobacterium shuttle plasmids and expressed in *trans*. Immobilization of specific antigens to the surface of the MBB was achieved by creating a fusion with the PHA synthase which remains covalently attached to the polyester core, resulting in PHA biobeads displaying covalently immobilized antigens. MBB, A:E-MBB, and an M. smegmatis vector control (MVC) were used in a mouse immunology trial, with comparison to phosphate-buffered saline (PBS)-vaccinated and Mycobacterium bovis BCG-vaccinated groups. We successfully produced MBB and A:E-MBB and used them as vaccines to induce a cellular immune response to mycobacterial antigens.

**IMPORTANCE** Tuberculosis (TB) is a disease caused by Mycobacterium tuberculosis or Mycobacterium bovis and still remains one of the world's biggest global health burdens. In this study, we produced polyhydroxyalkanoate (PHA) biobeads in mycobacteria and used them as vaccines to induce a cellular immune response to mycobacterial antigens.

## INTRODUCTION

Tuberculosis (TB) is a major cause of morbidity and mortality worldwide. The latest World Health Organization (WHO) global tuberculosis report estimated the occurrence of 10.4 million new TB cases worldwide and approximately 1.4 million TB deaths in 2015 (1). Current TB control strategies employ a partially effective live attenuated vaccine, Mycobacterium bovis bacillus Calmette-Guérin (BCG) (2), for the prevention of severe cases of TB in children and use of multiple antituberculosis drugs for the treatment of TB. These strategies have been successful in reducing both the incidence and the prevalence of TB globally. However, the emergence of TB caused by multidrug-resistant (MDR-TB) and extensively drug-resistant (XDR-TB) strains of M. tuberculosis has complicated control efforts (3, 4). Furthermore, the burden of HIV-associated TB (HIV-TB) remains problematic and accounts for a large proportion (25%) of all TB-related deaths (1, 5).

BCG vaccine is a live attenuated strain of M. bovis that was developed in the early 20th century for the prevention of TB globally, and to date BCG still remains the only available TB vaccine on the market. BCG, however, is seen to protect only against severe forms of childhood TB (tuberculous meningitis and miliary TB) and confers variable protection against pulmonary TB in adolescents and adults (2, 6, 7). Although BCG vaccination is regarded as safe, complications can occur in immunocompetent individuals. Adverse events linked to BCG vaccination range from mild, localized complications to more serious, systemic, or disseminated BCG disease in which M. bovis BCG is confirmed in one or more anatomical sites far from either the site of injection or regional lymph nodes. Furthermore, immunocompromised individuals such as those with HIV infection have a significantly higher risk of developing BCG-related diseases (8–11).

Consequently, there is great interest in new and novel vaccines that can offer better protection than the current BCG vaccine and that can also confer protection from TB in HIV-infected individuals. Currently, significant efforts are being made to develop new and improved vaccines against TB, with 15 candidate vaccines in clinical trials and more than 20 vaccines in early development (6). However, a limited understanding of immunity to M. tuberculosis is significantly hindering vaccine development (12).

Particulate vaccine delivery systems offer an advantageous approach to vaccine development. These systems are useful as they mimic various properties of pathogens, with size being the most important factor (13, 14). Further advantages of particulate delivery systems include the potential to target antigen-presenting cells (APC), to control antigen release, to display multiple antigens, and to include immunomodulatory molecules (15–17). Furthermore, particulate vaccine delivery systems can enhance the immune response as they demonstrate adjuvanting properties (18, 19) by promoting the uptake and trafficking of antigens to the local lymph nodes, which is a key step in the generation of potent immune responses. Immune response and antigen uptake by APCs can be influenced by a range of factors such as size, shape, surface charge, and solubility of the particulate delivery system used (13). Studies have shown that protective cellular immune responses are preferentially induced when antigens are displayed on small particulates such as virus-like particles (VLPs) (20), liposomes (20), immunostimulating complexes (ISCOMs) (21), chitosan (22), polylactide coglycolide (PLG) microparticles (17), and polyhydroxyalkanoate (PHA) biobeads (14, 16, 23).

PHA biobeads are of particular interest and offer an exciting new avenue for vaccine design. In comparison to other particulate systems, PHA biobeads offer two distinct advantages: (i) vaccine antigens are covalently bound in uniform direction to the surface, and (ii) these antigen-displaying beads can be produced in a one-step process (23, 24).

The most commonly found PHA is polyhydroxybutyrate (PHB), which requires three key enzymes, β-ketothiolase, acetoacetyl-coenzyme A (CoA) reductase, and PHA synthase (encoded by *phaA*, *phaB*, and *phaC*, respectively), for PHA biobead formation (25, 26).

Recently, our group demonstrated the use of PHA biobeads with surface immobilized antigens produced in Escherichia coli as safe and efficient vaccine delivery agents (23). These PHA biobeads were engineered to display M. tuberculosis vaccine candidate antigen 85A (Ag85A) and early secreted antigenic target 6-kDa protein (ESAT-6) by creating a gene fusion with the PHA synthase gene and expressing it in E. coli production hosts.

As an alternative to E. coli, a number of other bacteria, such as Gram-positive Lactococcus lactis, have been explored as possible production hosts (14, 27). PHA biobeads produced from both E. coli and L. lactis and displaying mycobacterial proteins Ag85A and ESAT-6 were tested as vaccines in mice and were found to induce significant protection mediated by Th1 and interleukin 17A (IL-17A)-biased T cell responses (16).

During these experiments, we found that bacterial host cell proteins were attached to the surface of the partially purified PHA biobeads, and we hypothesize that they too might function as antigens. If produced in mycobacteria instead of E. coli or L. lactis, such PHA biobeads should carry mycobacterial antigens on their surface, including not only known antigens but also many as yet undiscovered antigens that have the potential to induce protective immunity (28).

The aim of this study was to investigate the hypothesis that PHA biobeads can be produced in mycobacteria and function as vaccines protecting against mycobacterial infection. Therefore, we engineered Mycobacterium smegmatis as a model organism for the production of antigen-displaying PHA biobeads presenting the fusion protein Ag85A-ESAT-6 (16, 23). These PHA biobeads were expected to include host cell proteins derived from M. smegmatis that might serve as additional antigens, enhancing the induction of protective immunity and/or contributing adjuvanting properties. Mouse vaccinations were performed to assess the efficacy of these PHA biobeads.

Mycobacterial PHA biobeads potentially provide a new vaccination platform combining a large antigenic repertoire (comparable to that of live vaccines) with high safety (noninfectious vaccine and the absence of any genetic material) and ease and cost efficiency of production.

## RESULTS

### Production of MBB.

Several strategies have been explored for the establishment of the PHB pathway (genes *phaCAB*) in M. smegmatis. Initially, a two-plasmid system utilizing compatible E. coli-Mycobacterium shuttle plasmids pMycVec1 and pMycVec2 (29) was used, as was a system based on the pMIND plasmid (30, 31). However, both systems failed to produce PHB or detectable PhaC protein when *phaC* was expressed under a nitrile-inducible promoter (pNit) or a tetracycline-inducible promoter (pTet), respectively. A detailed description of these attempts is provided in the supplemental material.

The functionality of the pNit promoter was shown by transforming M. smegmatis with the plasmid pNit-1::*gfp* (31), carrying an ext-*gfp* reporter. Induction resulted in intense fluorescence (see Fig. S1A in the supplemental material), and the presence of green fluorescent protein (GFP) was confirmed by SDS-PAGE and immunoblotting (Fig. S1B and C). Therefore, plasmid pMV261 was engineered for the production of PhaC regulated under pNit.

**FIG 1:**
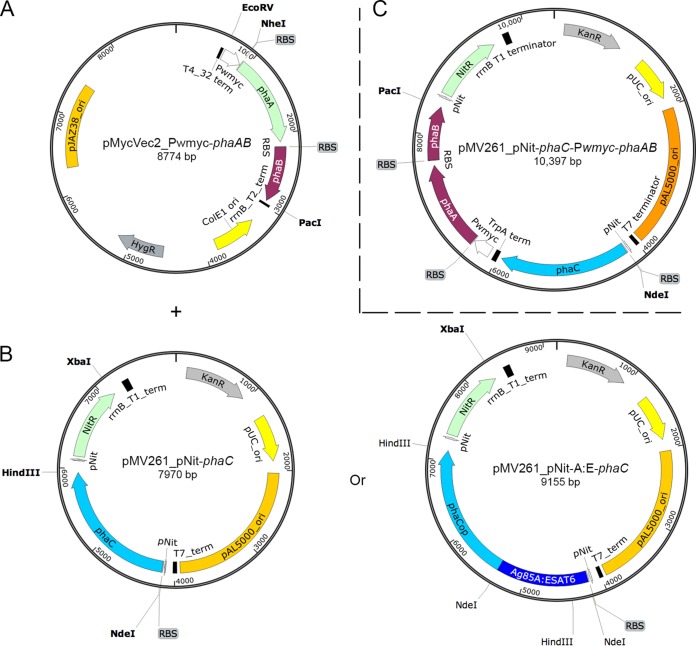
Two-plasmid and one-plasmid systems for PHB expression in mycobacteria. The two-plasmid system requires coexpression of plasmid pMycVec2_P*wmyc-phaAB* (carrying genes *phaAB* regulated under a weak constitutive mycobacterial promoter for synthesis of PHB precursor) (A) and plasmid pMV261_pNit-*phaC* (encoding PHA synthase) or pMV261_pNit-A:E-*phaC* (encoding fusion protein with Ag85A-ESAT-6) regulated under a nitrile-inducible promoter, pNit. (C) The one-plasmid system carries *phaC* regulated under pNit and genes *phaAB* under P*wmyc*. All restriction sites depicted are singular. Kan^r^ and Hyg^r^ confer resistance to kanamycin and hygromycin, respectively. Origins of replication in E. coli and mycobacterium are labeled. Transcription terminators are indicated by black bars.

**FIG 2:**
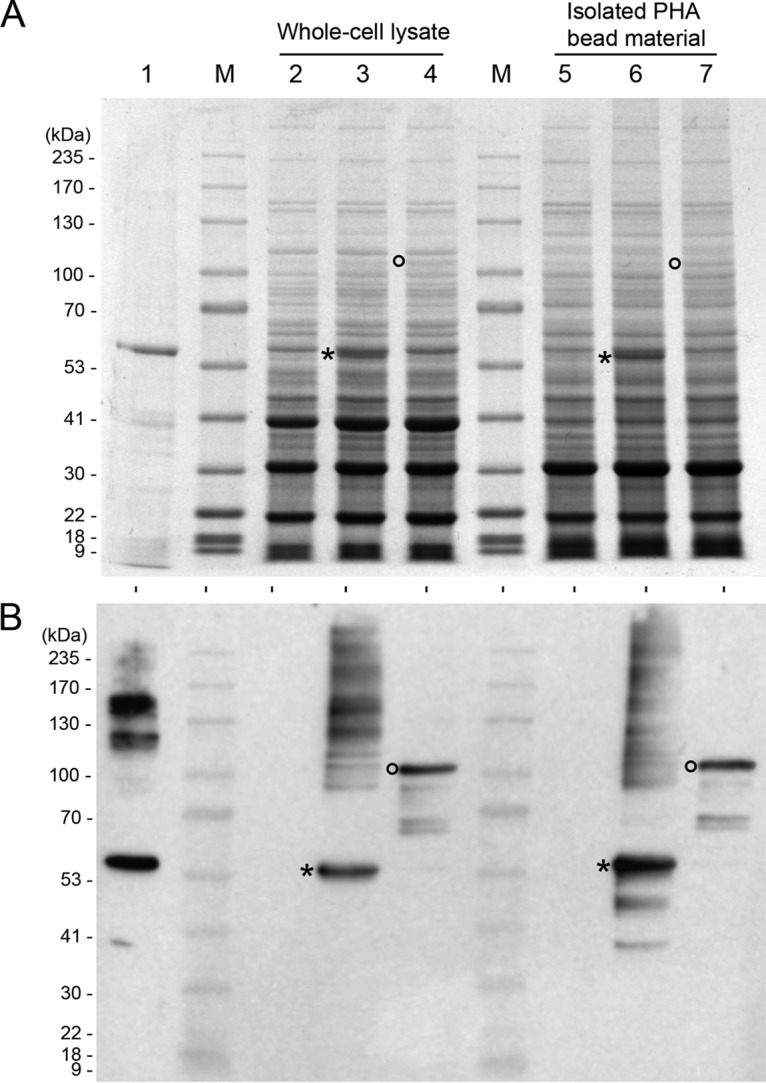
SDS-PAGE and immunoblot analysis of proteins from whole-cell lysate and isolated mycobacterial PHA biobead material. (A) SDS-PAGE with Coomassie blue staining; (B) immunoblot with anti-PhaC polyclonal antibodies. M. smegmatis whole-cell lysates: lane 1, positive control E. coli BL21-derived PhaC PHA biobeads; lane M, molecular weight standard; lane 2, pMycVec2_P*wmyc-phaAB* (MVC) negative control; lane 3, pMycVec2_P*wmyc-phaAB* and pMV261_*phaC* (MBB); lane 4, pMycVec2_P*wmyc-phaAB* and pMV261_A:E-*phaC* (A:E-MBB) and isolated mycobacterial PHA biobead material from M. smegmatis: lane 5, MVC; lane 6, MBB; lane 7, A:E-MBB. Asterisk, PhaC protein; circle, A:E-PhaC protein.

The mycobacterial PHA biobead (MBB) vaccine was produced in M. smegmatis cotransformed with pMycVec2_P*wmyc-phaAB* and pMV261_pNIT-*phaC* (Fig. 1A and B) under PHB-accumulating conditions. Production of PHB was confirmed by gas chromatography-mass spectroscopy (GC/MS) (Table 1; see also Fig. S2 in the supplemental material). Cells were disrupted by a bead mill, and subsequently MBB were enriched on a glycerol gradient as described in Materials and Methods. This vaccine preparation was called MBB. PhaC protein was visualized by SDS-PAGE in whole-cell and in isolated PHA biobead material and was subsequently confirmed by immunoblotting (Fig. 2). With the same protocol, a vector control based on M. smegmatis transformed with pMycVec2_P*wmyc-phaAB* and an empty pMV261_pNit was produced. This preparation was called the M. smegmatis vector control (MVC) and contains no PHB and hence no PHA biobeads.

**TABLE 1:** PHB biosynthesis of M. smegmatis harboring various plasmids

Plasmid^*a*^	PHB (% [wt/wt])^*b*^
No plasmid^*c*^	ND^*d*^
pMV261_pNit-*phaC*^*e*^	<1
pMV261_pNit-*phaC* + pMycVec2_P*wmyc-phaAB*^*c*^	5.2
pMV261_pNit-*phaC*-P*wmyc-phaAB*^*f*^	1.2
pMV261_pNit-A:E-*phaC* + pMycVec2_P*wmyc-phaAB*^*e*^	<1

a M. smegmatis mc^2^155 strain harboring various plasmids (one-plasmid or two-plasmid systems) grown under PHB-accumulating conditions as described in Materials and Methods.

b PHB yield is expressed as the percentage of PHB per milligram dry weight of whole cells calculated from known PHB standards.

c Experiments were conducted in triplicate, and the mean values are shown. The SD was <0.9.

d ND, not determined.

e Value shown from a single measurement.

f Experiment was conducted in duplicate, and the mean value is shown. The SD was 0.05.

In addition to the two-plasmid system, a one-plasmid system (pMV261_pNit-*phaC-Pwmyc-phaAB*) (Fig. 1C) containing codon-optimized genes *phaCAB* utilizing the backbone of vector pMV261 was created as a comparison. The resulting yield was 1.2% (wt/wt) PHB per cell dry weight (CDW) as shown by GC/MS (Table 1 and Fig. S2).

### Production of A:E-MBB.

Once stable expression of MBB was achieved, genes for a fusion protein consisting of the M. tuberculosis antigens Ag85A and ESAT-6 were engineered for display on the MBB surface (Fig. 1B). A previously developed gene fusion product of Ag85A-ESAT-6 (23) was attached to the 5′ end of *phaC*, resulting in plasmid pMV261_pNit-A:E-*phaC*. Expression of pMV261_pNit-A:E-*phaC* and pMycVec2_P*wmyc-phaAB* as a two-plasmid system (Fig. 1A and B) in M. smegmatis resulted in formation of A:E-MBB in these cells. The resulting yield of less than 1% (wt/wt) PHB per CDW as indicated by GC/MS (Table 1 and Fig. S2) was lower than that for the MBB. The presence of the fusion protein in these preparations was confirmed by immunoblotting with an ESAT-6-specific antibody (see Fig. S3 in the supplemental material). MBB were produced according to the same protocol as that for the MBB and MVC, and this third vaccine preparation was called A:E-MBB (mycobacterial PHA biobeads displaying Ag85A-ESAT-6).

### TEM analysis.

**FIG 3:**
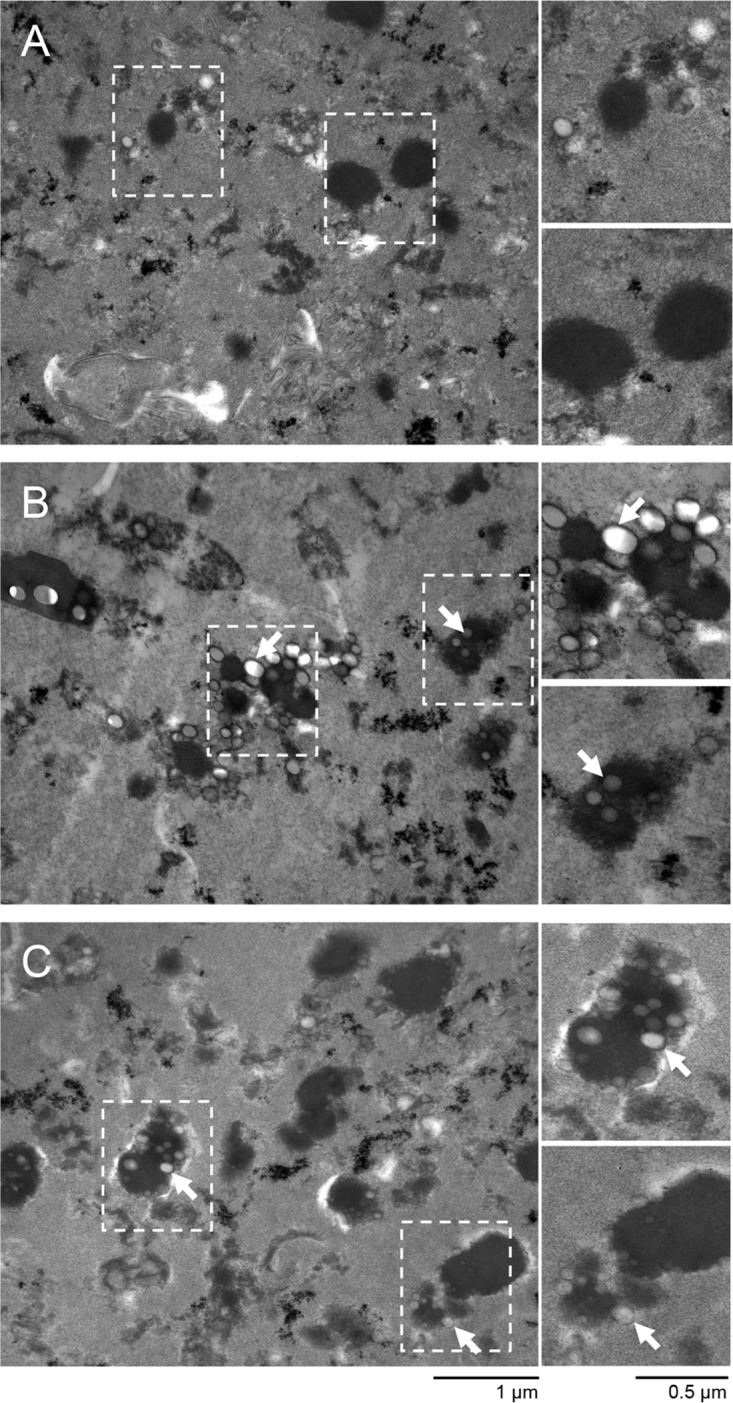
TEM analysis of isolated mycobacterial PHA biobead material by density gradient. Biobeads recovered from the 66%/90% interface of a glycerol gradient were subjected to TEM analysis. (A) MVC; (B) MBB; (C) A:E-MBB. All samples contain cellular debris and possible lipophilic inclusions. Mycobacterial PHA biobead-isolated material contains a large number of spherical inclusions of variable size, which is often localized with an unknown electron-dense staining material. White arrows indicate mycobacterial PHA biobeads.

Isolated MBB materials (MBB and A:E-MBB) and vector control material (MVC) were analyzed by transmission electron microscopy (TEM) (Fig. 3). Abundant small circular inclusions typical of PHA biobeads could be seen in the MBB and A:E-MBB preparations. Interestingly, these inclusions tend to be clustered around electron-dense staining material. Seemingly, more of these inclusions were observed in the MBB preparation than in A:E-MBB. Some circular inclusions were seen in the MVC preparation (Fig. 3A) and may suggest possible isolation of lipophilic inclusions typically produced in mycobacteria (32) in the MBB and A:E-MBB preparations (Fig. 3B and C). In addition to possible lipophilic inclusions, large amounts of cellular debris were seen in all preparations.

### MBB vaccination.

**FIG 4:**
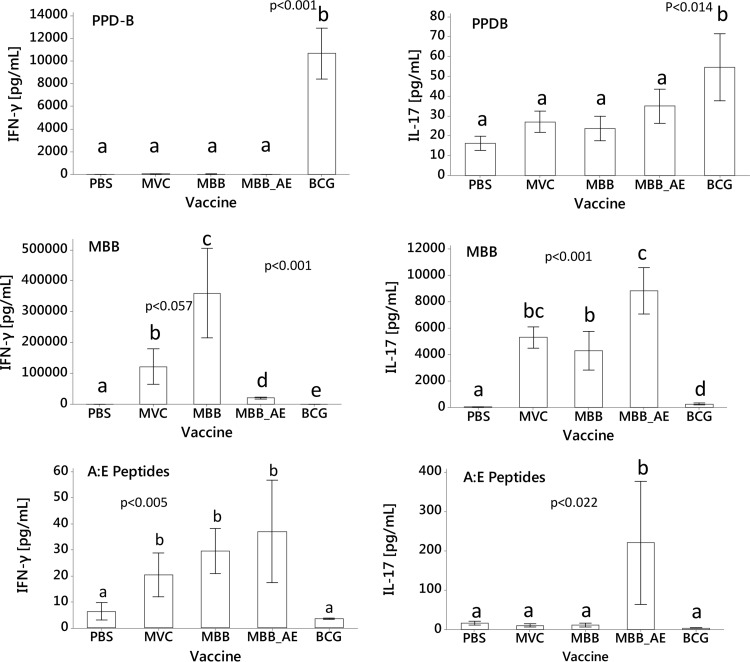
Cytokine responses from vaccinated mice. Vaccines were produced as described in Materials and Methods. Mice were vaccinated with either PBS, MVC, MBB, A:E-MBB (MBB_AE), or BCG. Seven weeks after the first immunization, mice were euthanized, and splenocytes were stimulated *in vitro* with PPD-B, MBB, or Ag85A-ESAT-6 (A:E) peptides. The amounts of secreted IFN-γ (left) and IL-17 (right) were determined by ELISA (*n* = 7 per vaccine group; mean ± SEM). Means with the same letters are not significantly different from each other based on analysis by one-way ANOVA. The ANOVA assumptions (normality, homogeneity of variances, etc.) were examined using model residuals and fitted values via diagnostic graphs and Shapiro's test for normality and Levene's test for homogeneity of variances.

Spleen cells from mice vaccinated with MBB showed strong gamma interferon (IFN-γ) responses when stimulated *in vitro* with MBB. These were significantly higher than all other responses (*P* < 0.057 for MVC; *P* < 0.001 for PBS, A:E-MBB, and BCG) (Fig. 4) and approximately 33 times higher than the response from BCG-vaccinated animals against PPD-B (M. bovis purified proteins). IFN-γ responses from the group vaccinated with A:E-MBB, however, were much lower but still approximately 3 times higher than the response of BCG-vaccinated animals against PPD-B. IFN-γ release in the MVC group to MBB was also very high. Animals vaccinated with vaccines generated in M. smegmatis, including the MVC, showed very strong IL-17 responses when stimulated with MBB. These were significantly higher than the responses from the PBS and BCG groups (*P* < 0.001) (Fig. 4) and approximately 90 to 160 times higher than the response from BCG-vaccinated animals against PPD-B. For IL-17, the A:E-MBB-vaccinated group showed significantly increased amounts of IL-17 compared to those for the PBS, MBB, and BCG groups (*P* < 0.001) but not the MVC.

Spleen cells from mice vaccinated with peptides showed higher IFN-γ secretion (Fig. 4) when restimulated *in vitro* with MVC, MBB, or A:E-MBB (*P* < 0.005) than with PBS or BCG. Also, IL-17 secretion from the A:E-MBB-vaccinated group increased when restimulated with Ag85A and ESAT-6 peptides compared to that for the other vaccinated groups (*P* < 0.022), indicating a strong peptide-specific response.

## DISCUSSION

The use of PHA biobeads produced in bacteria as a particulate vaccine delivery system engineered to display vaccine candidates against diseases such as tuberculosis has been previously shown (14, 16, 23, 33). The production of these PHA biobeads utilizes engineered heterologous hosts such as E. coli and L. lactis by establishing the pathway for PHB production. Despite subsequent enrichment by a glycerol gradient, such PHA biobeads can carry and display host cell proteins. These impurities can induce immune responses (14, 34). Mycobacterial cell wall (e.g., peptidoglycan, glycolipids, and mycolic acids) and intracellular (e.g., heat shock proteins and CpG) components are known to be responsible for the immunoadjuvant effect of Freund's complete adjuvant (FCA) (19). FCA is a water-in-oil emulsion containing inactivated M. tuberculosis and is a well-known potent stimulator of cell-mediated immunity (19).

In this study, we engineered M. smegmatis as a model organism for M. tuberculosis with the aim of producing mycobacterial PHA biobeads (MBB) and MBB displaying Ag85A-ESAT-6 (A:E-MBB). With production of PHA biobeads in a related rather than a nonrelated host, the copurified host compounds resemble that of the disease-causing pathogen. These compounds provide pathogen-specific adjuvant effects and as yet unidentified antigens that together are potential inducers of protective immunity.

Here, the PHB biosynthesis pathway (genes *phaCAB*) was successfully established in the host M. smegmatis. Members of the Mycobacteriaceae family do not naturally produce PHA, although putative synthases have been identified, e.g., MT1723 from M. tuberculosis. Establishment of the PHB biosynthesis pathway was difficult, and several strategies had to be explored to accomplish it.

The initial two-plasmid system strategy involved expressing *phaC* under a strong nitrile-inducible promoter (pNit) (31) and *phaAB* under a weak constitutive mycobacterial promoter (P*wmyc*) in compatible pMycVec1 and pMycVec2 vectors, respectively. However, expression and cultivation under PHB-accumulating conditions failed to produce detectible PHB due to the absence of recombinant PhaC protein being produced after induction. Similar results were obtained when green fluorescent protein (GFP) was being expressed under the same expression system in vector pMycVec1. Therefore, it was thought that the lack of recombinant protein production was due to a dysfunctional pNit promoter. Consequently, an alternative pMIND plasmid utilizing a tetracycline-inducible promoter (pTet) (30) was utilized for the expression of *phaC*. However, similarly, no PhaC protein was detected under inducing and PHB-accumulating conditions in M. smegmatis. Subsequent analysis was able to confirm that the pTet promoter was functional but only when expressed in *trans* in E. coli BL21 (see Fig. S4 in the supplemental material). This implies that pTet was not functional in M. smegmatis for unknown reasons.

The functionality of the pNit promoter was later confirmed after further analysis utilizing the GFP reporter protein in M. smegmatis only when expressed on the vector pMV261 (Fig. S1). Comparison of the dysfunctional vector pMycVec1 with pMV261 suggested that the absence of PhaC protein with pMycVec1 plasmid was due to a certain inherent property of the noncoding regions of pMycVec1. There is little difference in the coding regions of the two plasmids as they share the same origin of replication, selectable marker, and promoter. Therefore, *phaC* on pMV261 was designed to be expressed under the pNit promoter and used as a two-plasmid system with plasmid pMycVec2_P*wmyc-phaAB* that harbors the *phaAB* genes regulated under the *Pwmyc* promoter. For the hypothetical advantage of having a simpler production system, a single plasmid system version was also developed on the pMV261 backbone.

GC/MS analysis (Table 1) of recombinant M. smegmatis harboring PHB genes demonstrated that the two-plasmid system was better than the one-plasmid system and accumulated 4.3 times more PHB *in vivo*. The two-plasmid system was subsequently used for the production of MBB and A:E-MBB.

Recombinant M. smegmatis producing MBB was able to accumulate 5.2% (wt/wt) CDW (Table 1), which is comparable to recombinant PHB production in L. lactis (27), but less than what can be achieved with recombinant E. coli (up to 80% [wt/wt] CDW) (27, 35). The production of A:E-MBB, however, resulted in substantially less PHB (<1% [wt/wt] CDW). Both the PHA synthase (PhaC) and fusion protein variant (A:E-PhaC) were not found to be overproduced in M. smegmatis using the current pNit gene expression system (Fig. 2).

Furthermore, it was found that translational fusion of Ag85A-ESAT-6 to the N terminus of PhaC seems to impact negatively on recombinant protein production compared to that for PhaC alone. Other studies have shown translational fusions to PhaC impacted PHA accumulation *in vivo*, and this variation seems to be strongly dependent on the PHA synthase fusion partner (35–37). Further improvements to protein production and PHB yield need to be explored.

The ability to isolate PHA biobeads from non-biobead-associated host cell debris was a limiting factor in this study. Complete lysis and isolation of mycobacterial PHA biobeads proved challenging due to the thick and waxy cell wall properties of mycobacteria (38). SDS-PAGE analysis (Fig. 2A) of the whole-cell and isolated material showed minor differences in protein profiles, suggesting a substantial amount of host cell impurities in the isolated PHA biobead material, which is reflected with TEM (Fig. 3B and C). These impurities might include cell wall debris, mesosomes (39), and possibly non-PHA intracellular lipophilic inclusions (32, 39). The PHA biobeads were often colocalized with electron-dense staining bodies of unknown origin and function. In the future, further improvements to the isolation protocol need to be explored to improve recovery of MBB and removal of non-biobead-associated impurities.

To gain a preliminary understanding of the immunogenicity of MBB and A:E-MBB and also to evaluate the hypothesis that copurified mycobacterial antigens contribute to the immune response, these biobeads were used in a mouse vaccination study. Cytokines secreted by *in vitro*-stimulated splenocytes from vaccinated animals and disease progression were analyzed. The high degree of copurified material in the vaccine preparations made the subsequent interpretation of the immunological responses difficult. It is likely that most of the immune responses measured were induced by these materials, which were also present in the vector control. These materials have likely triggered both innate and adaptive immune responses, leading to much stronger cytokine responses than those induced by highly purified biobead preparations used in past studies (16). However, the finding of a strong IL-17 response to the recombinant peptides encourages the interpretation that A:E-MBB can be further developed into an effective vaccine. This future work will need to use vaccine preparations with a much higher degree of purity that contain only antigens displayed on the surface of biobeads. To include yet more relevant antigens, it was also produced in BCG, M. bovis, and M. tuberculosis. Provision of the cloning strategy for expression in these hosts was the reason for choosing M. smegmatis as the production host in this study.

The cytokine IFN-γ has long been known to play a major role in protection against tuberculosis. More recently, IL-17 has been identified as a further key cytokine for control of tuberculosis (40, 41). Hence, supernatants obtained after *in vitro* restimulation were analyzed for secretion of IFN-γ and IL-17 using an enzyme-linked immunosorbent assay (ELISA).

All mice vaccinated with PHA biobeads derived from mycobacteria, including the MVC, showed strong IFN-γ and IL-17 responses when stimulated with MBB (Fig. 4). These responses were stronger than the response from BCG-vaccinated animals against PPD-B (Fig. 4). Mice vaccinated with MBB showed the strongest IFN-γ response when stimulated with MBB, which was higher than all other responses. Compared to MBB vaccinates, IFN-γ responses from the group vaccinated with A:E-MBB were much lower. This lower response is likely due to the low concentration of PHA biobeads in these preparations or replacement or masking of epitopes on the PHA biobead surface by the displayed Ag85A-ESAT-6.

A strong adjuvant effect of M. smegmatis-derived material “contaminating” the vaccine is a probable cause for the high IFN-γ release shown by the MVC-vaccinated control group. This supports our hypothesis that contaminating material originating from the pathogen can stimulate an immune response. Future work will have to be performed to investigate what amount of contaminating material leads to an optimal pathogen-specific immune response.

IL-17 has been proposed as being important for protection by mediating the recruitment of neutrophils and promoting the entry of Th1 cells to the site of granuloma formation (42). The A:E-MBB-vaccinated group showed significantly increased secretion of IL-17 compared to that of the other groups when stimulated with MBB (Fig. 4, bottom row, right graph). As mice vaccinated with MVC also showed a strong IL-17 response, it is likely that the IL-17 response was due to the host cell impurities in the MBB and A:E-MBB preparations.

This study used MBB produced in M. smegmatis, which was chosen over M. tuberculosis and M. bovis for its faster growth in culture, leading to faster production of MBB. However, M. smegmatis and M. tuberculosis and M. bovis differ significantly genetically (43, 44). M. smegmatis lacks a large proportion of proteins found in the pathogenic strains (45). Hence, the immune response to impurities of M. smegmatis is likely to be less effective in conferring protection against pathogenic strains such as M. bovis. The strategy of engineering M. bovis or M. tuberculosis for the production of MBB or A:E-MBB may consequently offer better vaccine efficacy.

The use of TB antigen displaying biobeads produced in E. coli in a heterologous prime-boost strategy has been previously investigated (33). Biobead vaccines can contain a large repertoire of compounds that have the potential to increase the effect of the boost vaccine (46). However, future studies will have to be preformed to investigate the adjuvant effects of copurified host compounds by comparison with highly purified vaccine preparations. We propose that mycobacteria-derived biobeads may be advantageous for use in a heterologous prime-boost strategy as a prime and/or boost. Studies have shown that a heterologous prime-boost strategy is more protective than a homologous prime-boost strategy (47–49).

In conclusion, this study proves the feasibility of the production of PHA biobeads in mycobacteria and also provides preliminary insights into their efficacy as vaccines against tuberculosis. Future studies should include improvements in the mycobacterial PHA biobead production process, production of PHA biobeads in M. bovis and/or M. tuberculosis, and inclusion of additional or alternative mycobacterial antigens. More detailed immunological and challenge trials should be undertaken on these new preparations.

To summarize, we have introduced a promising new vaccination platform potentially combining defined and unknown copurified antigens (comparable to live attenuated or inactivated vaccines) with high safety (noninfectious vaccine and absence of any genetic material) and ease and cost efficiency of production.

## MATERIALS AND METHODS

### Bacterial strains and cultivation conditions.

Bacterial strains used in this study are listed in Table 2. E. coli strains were cultivated in Luria broth (Difco, Detroit, MI). For LB agar, 1.5% (wt/vol) agar was added to liquid. M. smegmatis was cultivated on BBL Middlebrook 7H9 broth or 7H10 agar (BD, Franklin Lakes, NJ, USA) supplemented with 0.2% (vol/vol) glycerol and 10% (vol/vol) OADC (BD). Additionally, 7H9 broth was supplemented with 0.05% Tween 80 (vol/vol).

**TABLE 2:** Bacterial strains, plasmids, and oligonucleotides used in this study

Strain, plasmid, or oligonucleotide	Description^*a*^	Reference or source
Strains		
E. coli		
XL1-Blue	*recA1 endA1 gyrA96 thi-1 hsdR17 supE44 relA1 lac* [F′ *proAB lacI*^q^ *lacZ*ΔM15 Tn*10* (Tet^r^)]	Stratagene
BL21(DE3)	E. coli B F^−^ *dcm ompT hsdS*(r_B_^−^ m_B_^−^) *gal* λ(DE3)	Stratagene
Mycobacterium smegmatis		
mc^2^155	Ept^−^, Kan^s^	53
Plasmids		
pMycVec1	E. coli-Mycobacterium vector, pAL5000 origin, ColE1 origin (pBR322); Kan^r^	29
pMycVec1_pNit-*phaC*	pNit promoter and *phaC* from pET-16b_pNit-*phaC*	This study
pMycVec2	pMS2 cloning vector for mycobacteria, pJAZ38 origin, ColE1 origin (pUC19); Hyg^r^	This study
pMycVec2_P*wmyc-phaAB*	Amplified *phaAB* regulated under constitutive promoter P*wmyc*	This study
pMIND	E. coli-Mycobacterium vector, pAL5000 origin, ColE1 origin; pTetRO; Kan^r^; Hyg^r^	30
pMIND_pTet-*phaC*	M. tuberculosis codon usage optimized *phaC* regulated under *tetRO* operator	This study
pMV261_pNit	E. coli-Mycobacterium vector with pNit promoter, pAL5000 origin, pUC origin; Kan^r^	This study
pNit-1::*gfp*	pMV261 containing ext-*gfp* reporter gene regulated under pNit promoter	31
pMV261_pNit-*phaC*	*phaC* from pUC57_*phaC* regulated under pNit	This study
pMV261_pNit-A:E-*phaC*	Ag85A-ESAT-6 hybrid gene upstream of *phaC* regulated under pNit	This study
pMV261_pNit-*phaC*-P*wmyc-phaAB*	pMV261_pNit-*phaC* derivative containing gene fragment *phaCAB* from pUC57_pTet-*phaC*-P*wmyc-phaAB*_PacI	
pET-16b	Ap^r^, T7 promoter	Novagen
pET-16b_pNit	pET-16b derivative containing *gfp* regulated under pNit promoter	This study
pET-16b_pNit-*phaC*	*phaC* derived from pUC57_*phaC*	This study
pMCS69	pBBR1MCS derivative containing *phaAB* genes from Cupriavidus necator	50
pHAS-Ag85A-ESAT-6	pHAS containing Ag85A-ESAT-6 hybrid gene upstream of *phaC*	23
pGEM-T_P*wmyc*	Carrying a synthesized weak constitutive promoter (P*wmyc*) from M. smegmatis	This study
pUC57_*phaC*	Synthesized *phaC* from Cupriavidus necator codon usage optimized for expression in M. tuberculosis	This study
pUC57-*phaC*-P*wmyc-phaAB*	Synthesized *phaC* regulated under *tetRO* operator and genes *phaAB* regulated under weak constitutive M. smegmatis promoter P*wmyc*; all gene codon usage optimized for expression in M. tuberculosis	This study
pUC57-*phaC*-P*wmyc-phaAB*_PacI	Same as pUC57_*phaC*-P*wmyc-phaAB* but with the introduction of PacI restriction site downstream of *phaB*	This study
pUC57_3′*phaB*_PacI	Carrying synthesized gene fragment for the introduction of PacI site to 3′ end of *phaB*	This study
Oligonucleotides (5′ to 3′)		
fwd_BamHI_pNit	ATAGGATCCAGGACCCTTGTCATTCCACGTCAATTC	This study
rev_pNit_XbaI	TGTCGTCATATCTAGACTACGAAACCTCCGTCGG	This study
fwd_NheI_phaAB	AAAGCTAGCAAGGAGTACACAATGACTGACGTTGTCATCG	This study
rev_phaAB_PacI	TATTTAATTAATCAGCCCATATGCAGGCCGCCGTTGAG	This study
fwd_A:E	ACATATGTTTTCCCGGCCGGGCTTG	This study
rev_A:E	TCATATGACTAGTTGCGAACATCCCAGTGACG	This study

a Tet^r^, tetracycline resistance; Kan^s^, kanamycin sensitivity; Kan^r^, kanamycin resistance; Hyg^r^, hygromycin resistance; Ap^r^, ampicillin resistance.

If required, antibiotics were added to the medium at the following concentrations: ampicillin, 100 μg/ml (E. coli); kanamycin, 30 μg/ml (E. coli and M. smegmatis); and hygromycin, 200 μg/ml (E. coli) or 90 μg/ml (M. smegmatis). Unless stated otherwise, E. coli strains and M. smegmatis mc^2^155 were cultivated at 37°C with aeration at 200 rpm. When required for protein induction, tetracycline or isovaleronitrile was added at a final concentration of 30 ng/ml or 50 μm/ml, respectively.

### Construction of plasmids for the production of PHB.

All plasmids and oligonucleotides used can be found in Table 2. All synthesized genes have been codon usage optimized for M. tuberculosis. DNA sequencing was used to confirm all amplified and final plasmids. Several different plasmids were generated for the expression of PHA synthase alone (*phaC*) or as a fusion protein with antigens Ag85A-ESAT-6 utilizing either the nitrile-inducible (pMycVec1 and pMV261 derivatives) or the tetracycline-inducible (pMIND derivatives) promoter. A single plasmid was generated for the expression of PHB precursor genes (*phaAB*) regulated under a weak constitutive M. smegmatis promoter (P*wmyc*).

A one-plasmid system was also created which carried all three genes, with *phaC* regulated under the nitrile promoter (pNit) and genes *phaA* and *phaB* under P*wmyc* utilizing the pMV261 vector (31) for comparison with the two-plasmid system for protein and PHB production.

### (i) Plasmid pMycVec1_pNit-*phaC*.

This plasmid carries the gene for the class I PHA synthase (*phaC*) regulated under the strong nitrile-inducible promoter pNit (pNit-1::*gfp* was kindly provided by Chris Sassetti, University of Massachusetts, Worcester, MA). First, the nitrile promoter- and ext-*gfp*-carrying fragment was amplified from plasmid pNit-1::*gfp* (31) using primers fwd_BamHI_pNit and rev_pNit_XbaI. The resultant fragment was ligated into intermediate cloning vector pGEM-T Easy (Promega, Madison, WI, USA). Following confirmation, the pNit-*gfp* fragment was excised using BamHI and XbaI and successively ligated with vector pET-16b. NdeI and SwaI were used to replace the ext-*gfp*-carrying fragment with the M. tuberculosis codon-optimized gene *phaC* with complementary sites isolated from pUC57_*phaC*. The DNA fragment carrying pNit_*phaC* from plasmid pET-16b_pNit-*phaC* was then excised using BamHI and XbaI and subsequently ligated with E. coli-Mycobacterium shuttle plasmid pMycVec1.

### (ii) Plasmid pMycVec2_P*wmyc-phaAB*.

The E. coli codon usage-optimized genes *phaA* and *phaB* required for precursor synthesis in the two-plasmid system were amplified from plasmid pMCS69 (50) using primers fwd_NheI_phaAB and rev_phaAB_PacI and subsequently ligated into pGEM-T easy. The amplified fragment containing *phaA* and *phaB* was excised using NheI and PacI following confirmation by DNA sequencing. A synthesized DNA fragment containing the weak constitutive promoter P*wmyc* from M. smegmatis was excised from pGEM-T_P*wmyc* using EcoRV and NheI. DNA fragments carrying precursor genes (*phaA* and *phaB*) and P*wmyc* were used in a single ligation reaction with E. coli-Mycobacterium shuttle plasmid pMycVec2 linearized with EcoRV and PacI.

### (iii) Plasmid pMIND_pTet-*phaC*.

The DNA fragment carrying codon-optimized gene *phaC* was excised from plasmid pUC57_*phaCAB* using BamHI and HindIII. The fragment was subsequently ligated into the corresponding sites of plasmid pMIND (30) downstream of the tetracycline-inducible promoter (pTet).

### (iv) Plasmid pMV261_pNit-*phaC*.

Plasmid pNit-1::*gfp* was hydrolyzed with the NdeI and HindIII excising reporter ext-*gfp* to allow the ligation of codon-optimized gene *phaC* from plasmid pUC57_*phaC* into the corresponding sites.

### (v) Plasmid pMV261_pNit-A:E-*phaC*.

The DNA fragment encoding fusion antigens Ag85A-ESAT-6 was amplified from existing plasmid pHAS-Ag85A-ESAT-6 (23) using primers fwd_A:E and rev_A:E. The resultant fragment was then ligated into vector pGEM-T. Subsequently, the DNA fragment encoding Ag85A-ESAT-6 was excised using NdeI and ligated into the corresponding sites in plasmid pMV261_pNit-*phaC* located downstream of the pNit promoter and upstream of codon-optimized *phaC*.

### (vi) Plasmid pMV261_pNit-*phaC*-P*wmyc-phaAB*.

pMV261_pNit-*phaC*-P*wmyc-phaAB* is a single vector system utilizing vector pMV261. First, the PacI site was introduced downstream of *phaB* in plasmid pUC57_*phaCAB* using a small synthesized DNA fragment from plasmid pUC57_3′*phaB_*PacI carrying a short segment of the 3′ end of *phaB* and the PacI site. This allowed the subcloning of the entire DNA fragment carrying codon-optimized *phaC* and precursor genes (*phaA* and *phaB*) regulated under a weak constitutive mycobacterial promoter P*wmyc*. The fragment was excised from plasmid pUC57_3′*phaB_*PacI using XhoI and SpeI and inserted into the corresponding sites of plasmid pUC57_*phaC*-P*wmyc-phaAB*. Consequently, the DNA fragment encoding PHA synthase and carrying the precursor genes was excised from pUC57_*phaC*-P*wmyc-phaAB_*PacI using NdeI and PacI and ligated into the corresponding sites in vector pMV261_pNit-*phaC* downstream of the pNit promoter, resulting in the replacement of the existing *phaC* gene.

### PHB-accumulating growth conditions.

To assess the ability of M. smegmatis to accumulate PHB *in vivo*, strains were cultivated under PHB-accumulating conditions. Ten milliliters of 7H9 broth supplemented with 1% glucose (vol/vol) as the carbon source and antibiotics was inoculated with frozen glycerol stock cultures and cultivated for 20 h. Then 50 ml of fresh 7H9 broth supplemented with antibiotics was added to the preculture and cultivated for another 20 h. Large 1-liter cultures of 7H9 broth supplemented with antibiotics were inoculated with 3% (vol/vol) preculture and cultivated to an optical density at 600 nm (OD_600_) of 0.3 to 0.4. Once the OD_600_ was reached, protein expression was induced by the addition of isovaleronitrile at a final concentration of 50 μm and then further cultivation was performed for 72 h.

### Isolation of MBB.

Forty-eight-hour postinduction cultures were harvested by centrifugation at 9,000 × *g* for 20 min. Sediment was washed once and then suspended as a 25% slurry (wt/vol) with 1× PBS (pH 7.4). Then 1 ml of the 25% slurry was aliquoted into 2-ml screw-cap tubes containing 1/3 volume or 500 μl (vol/vol) acid-washed 0.1-mm glass beads and chilled on ice for 10 min prior to cell lysis. Lysis was achieved by using a Hybaid RiboLyser (FP120HY-230) with the following procedure: 6 m/s for 3 1-min intervals with 2-min cooling steps in between each interval. Lysates was separated from glass beads by centrifuging briefly at 3,000 × *g* for 10 s. Then 1 ml of the 25% lysate was loaded onto a glycerol gradient consisting of 44%/66%/90% (vol/vol) glycerol layers made in 1× PBS (pH 7.4). Separation was achieved by centrifugation at 100,000 × *g* for 1.5 h, and MBB were recovered from the 66% and 90% glycerol interfaces. Isolated MBB were washed two times using 1× PBS (pH 7.4) with centrifugation at 9,500 × *g* for 30 min and then formulated in 1× PBS (pH 7.4) as a 20% (wt/vol) slurry. To ensure sterile preparations, washed MBB were heat treated in sterile 2-ml screw-cap tubes at 80°C for 30 min. Sterility was confirmed by cell culture on antibiotic-free supplemented BBL Middlebrook 7H10 agar (BD).

### PHB analysis by GC/MS.

Lyophilized material (40 to 60 mg) was suspended in 2 ml of chloroform and subjected to methanolysis in 2 ml of methanol in the presence of 15% (vol/vol) sulfuric acid. Methanolysis was performed in a heated oil bath for 5 h at 100°C. After methanolysis, tubes were cooled to room temperature, 2 ml of distilled water was added, and the tubes were briefly vortexed. Samples were then left at room temperature for phase separation. The bottom phase, containing methyl esters of the corresponding fatty acid constituents, was recovered and analyzed by GC/MS for 3-hydroxyalkanoate methyl esters.

### TEM.

Transmission electron microscopy (TEM) analysis was used to assess isolated PHA biobead material produced in M. smegmatis. Samples were processed for analysis as described previously (51).

### Protein analysis.

Proteins were separated using SDS-PAGE and visualized by staining with Coomassie brilliant blue. Immunoblotting was used to confirm PhaC or Ag85A-ESAT-6-PhaC fusion protein on MBB or the production of GFP. Protein bands separated by SDS-PAGE were transferred to nitrocellulose membranes using the iBlot system (Invitrogen, Carlsbad, CA). Membranes were blocked with 1% skim milk in phosphate-buffered saline with Tween 20 (PBST) for 1 h. Following washing with PBST, primary antibodies were diluted in 1% bovine serum albumin (BSA) and used accordingly: for detection of PhaC, 1:20,000 rabbit polyclonal (GenScript, NJ); ESAT-6, 0.1 μg/ml rabbit polyclonal (Abcam, Cambridge, United Kingdom); and GFP, 0.75 μg/ml rabbit polyclonal (A01388; GenScript, NJ). Following incubation for 1 h, the membrane was washed three times with PBST for 5 min. Secondary antibody anti-rabbit horseradish peroxidase (HRP) at 1:25,000 (Ab6721; Abcam, UK) was diluted in 1% BSA, added, and incubated for 1 h. Following three PBST washes, development was carried out using SuperSignal West Pico chemiluminescent substrate (Thermo Fisher, Waltham, MA).

The BCA protein assay kit (23227; Thermo Scientific, IL) was used according to the manufacturer's instructions to quantify the total protein in the isolated MBB material.

### Mouse immunization trial.

To evaluate the efficacy of MBB as a vaccine for tuberculosis a mouse immunology trial was performed. All animal experiments were approved by the Grasslands Animal Ethics committee (approval number 13100; Palmerston North, New Zealand).

### Animals.

C57BL/6 female mice, aged 6 to 8 weeks at the start of the experiment, were obtained from the AgResearch Ruakura Small Animal Unit (Hamilton, New Zealand). The animals were assigned to one of five vaccination groups (12 animals per group) and housed at AgResearch's PC2 Ulyatt-Reid Small Animal Facility (Palmerston North, New Zealand). The animals were kept in a separate room of this PC2 facility but not under specific-pathogen-free conditions.

### Vaccination.

One control vaccination group received three biweekly, subcutaneous injections of PBS. Another control group received three biweekly, subcutaneous injections of M. smegmatis vector control (MVC) lysate, which did not contain any MBB. Two further groups were vaccinated with three biweekly, subcutaneous injections of either MBB or MBB displaying Ag85A-ESAT-6 (A:E-MBB). Each of these vaccine preparations was applied at 300 μg of total protein in 200 μl of PBS. Finally, mice in another control group (BCG) received a single dose of 10^6^ CFU of BCG Pasteur strain 1173P2 (kindly provided by the Malaghan Institute of Medical Research, Wellington, New Zealand).

### Cell-mediated immune response.

Seven weeks after the first immunization, seven mice from each group were euthanized and splenocytes were prepared for further analysis. Splenocytes were stimulated *in vitro* using various antigen preparations at a final concentration of 5 μg/ml: (i) PBS (as an unstimulated negative control), (ii) concanavalin A (ConA) (C-0412 [Sigma-Aldrich], a mitogen, used as a positive control [data not shown]), (iii) purified protein derivative from M. bovis (PPD-B) (Prionics AG, Switzerland), (iv) MBB, (v) peptides from Ag85A (amino acids [aa] 99 to 118, aa 145 to 152) and ESAT-6 (aa 1 to 16, aa 9 to 24, aa 17 to 32, aa 57 to 72, and aa 80 to 95), or (vi) PhaC peptides (aa 110 to 118 and aa 118 to 126). Supernatants from these cultures were harvested, and the amounts of secreted IFN-γ and IL-17 were determined by ELISA.

### Statistical analysis.

For the analysis of cytokine measurements from culture supernatants of restimulated splenocytes, one-way analysis of variance (ANOVA) was carried out with 5 treatments (vaccine levels) separately for IL-17 and IFN-γ and separately for *in vitro* restimulation with PPD-B, MBB, and peptide subsets. The ANOVA assumptions (normality, homogeneity of variances, etc.) were examined using model residuals and fitted values via diagnostic graphs and Shapiro's test for normality and Levene's test for homogeneity of variances. Transformations were required, and the log (natural) transformations gave reasonably satisfactory ANOVA assumptions. All analyses were carried out using R software, version 3.3.1 (52).

## Supplementary Material

Supplemental material
