# Supplementary material for: Engineering Mycobacteria for the Production of Self-Assembling Biopolyesters Displaying Mycobacterial Antigens for Use as a Tuberculosis Vaccine
Source: Appl Environ Microbiol. 2017 Feb 15;83(5):e02289-16. doi: 10.1128/AEM.02289-16 (PMC5311400; doi:10.1128/AEM.02289-16)
Supplement: Supplemental material [file supp_83_5_e02289-16__index.html]

Supplemental material 

# Engineering Mycobacteria for the Production of Self-Assembling Biopolyesters Displaying Mycobacterial Antigens for Use as a Tuberculosis Vaccine

## Supplemental material

- Supplemental file 1 -

  Supplemental results: production of MBB; confirmation of pNit promoter activity (Fig. S1); GC-MS analysis of PHB in *M. smegmatis* cells harboring various plasmids (Fig. S2); confirmation of ESAT-6 (Fig. S3); GC-MS analysis of PHB in *E. coli* BL21 cells harboring plasmids pMCS69 and pMIND\_pTet-*phaC* (Fig. S4).

  PDF, 2.2M
