# Supplementary material for: Engineering Mycobacteria for the Production of Self-Assembling Biopolyesters Displaying Mycobacterial Antigens for Use as a Tuberculosis Vaccine
Source: Appl Environ Microbiol. 2017 Feb 15;83(5):e02289-16. doi: 10.1128/AEM.02289-16 (PMC5311400; doi:10.1128/AEM.02289-16)
Supplement: Supplemental material [file AEM.02289-16_zam999117679s1.pdf]

Supplemental Material

Supplemental Results: Production of MBB

The initial strategy to establish the PHB pathway (genes *phaCAB*) in *M. smegmatis* was to apply a two-plasmid system utilizing compatible *E. coli−Mycobacterium* shuttle plasmids pMycVec1 and pMycVec2 (1). PhaC was designed to be expressed on the higher copy number plasmid pMycVec1 utilizing a strong nitrile-inducible promoter (pNit) (2) to promote high expression, while *phaAB* genes (3) required for the production of precursor (R)-3-hydroxybutyrate-CoA were selected to be expressed under a weak constitutive mycobacterial promoter (P*wmyc*) (1) on the low copy number plasmid pMycVec2, as the precursor is required in catalytic amounts. Mycobacterium codon-optimized gene and nonoptimized *phaAB* genes were used and all amplified DNA fragments and final plasmids were confirmed by sequencing. Unfortunately, expression of this two-plasmid system consisting of pMycVec1_pNit-*phaC* and pMycVec2_P*wmyc-phaAB* in *M. smegmatis* under PHB accumulating conditions did not result in PHB production. Results from SDS-PAGE and immunoblot with anti-PhaC antibodies suggest PhaC protein was not being produced. Similarly, GFP reporter was not detectable by SDS-PAGE and immunoblot with GFP specific polyclonal antibodies using the same pMycVec1 expression system with *gfp* gene regulated under the pNit promoter. This suggests a possible problem with the pMycVec1 expression system utilizing promoter pNit.

An alternative cloning strategy was designed based on the pMIND plasmid (2, 4) utilizing a weaker tetracycline inducible promoter (pTet) for the expression of the same codon-optimized *phaC* in *M. smegmatis*. Expression of pMIND_*phaC* in *M. smegmatis* as a two-plasmid system with pMycVec2_P*wmyc-phaAB* also failed to produce detectable PhaC protein or PHB. However, coexpression of pMIND_*phaC* in

E. coli BL21(DE3) and pMCS69 plasmid encoding *phaAB*, showed accumulation of 1.2% PHB per (wt/wt) cellular dry weight (CDW) in whole-cell samples analyzed by GC/MS (FIG. S4), indicating pMIND_*phaC* was functional. As to why pMIND_*phaC* was not functional in *M. smegmatis* is unknown.

1. **Kaps I, Ehrt S, Seeber S, Schnappinger D, Martin C, Riley LW, Niederweis M.** 2001. Energy transfer between fluorescent proteins using a co-expression system in *Mycobacterium smegmatis*. Gene **278:**115-124.
2. **Pandey AK, Raman S, Proff R, Joshi S, Kang CM, Rubin EJ, Husson RN, Sassetti CM.** 2009. Nitrile-inducible gene expression in mycobacteria. Tuberculosis **89:**12-16.
3. **Amara A, Rehm B.** 2003. Replacement of the catalytic nucleophile cysteine-296 by serine in class II polyhydroxyalkanoate synthase from *Pseudomonas aeruginosa*-mediated synthesis of a new polyester: identification of catalytic residues. Biochem J **374:**413-421.
4. **Blokpoel MC, Murphy HN, O'Toole R, Wiles S, Runn ES, Stewart GR, Young DB, Robertson BD.** 2005. Tetracycline-inducible gene regulation in mycobacteria. Nucleic Acids Research **33:**e22-e22.

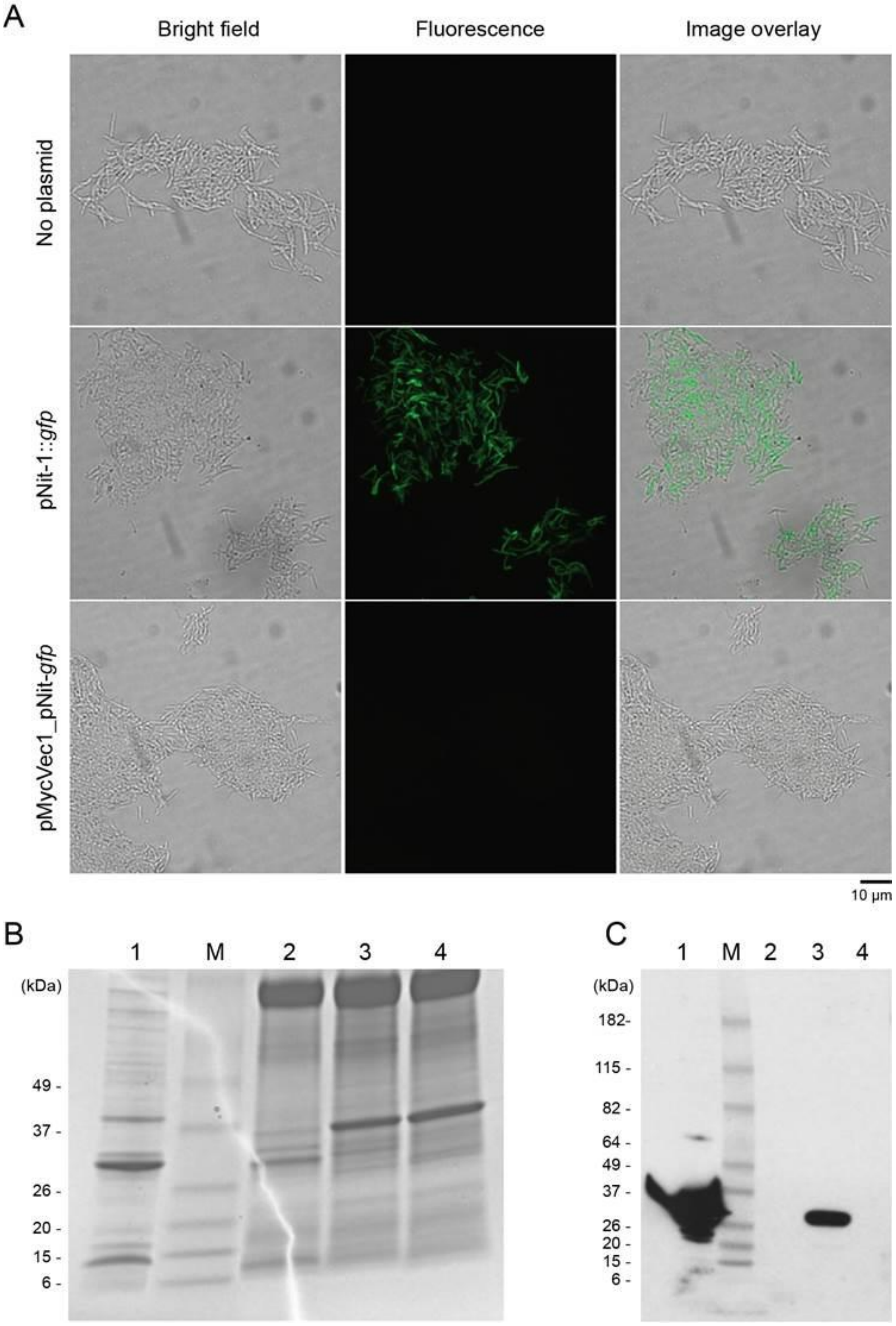

FIG. S1. Confirmation of pNit promoter activity. *M. smegmatis* harboring various plasmid systems encoding *gfp* regulated under nitrile inducible promoter (pNit) were grown under protein inducive conditions. (A) Fluorescent microscopy analysis of *M. smegmatis* without plasmid and with plasmid pNit-1::*gfp* for detection of GFP. Protein analysis was performed by (B) SDS-PAGE and (C) immunoblot analysis. Samples were arranged accordingly: lane 1, GFP positive control; lane M, molecular weight standard; lane 2, *M. smegmatis* with no plasmid; lane 3, pNit-1::*gfp*; and lane 4, pMycVec1_pNit-*gfp*.

Sample Name : *M. smegmatis mc[2]155*
Injection Volume : 1.00

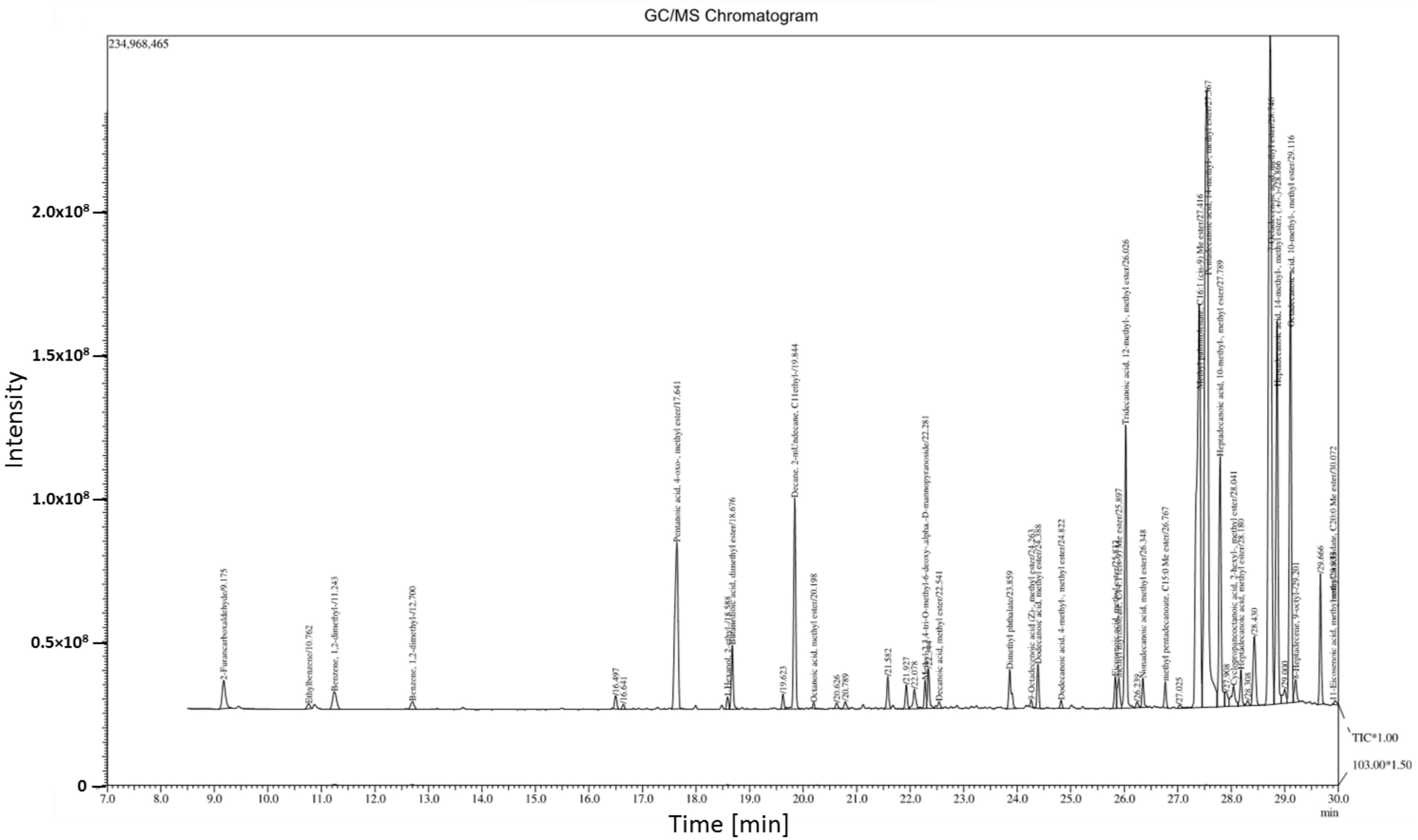

FIG. S2. Analysis of PHB in whole-cell by GC/MS. *M. smegmatis* harboring various plasmids were cultivated under PHB accumulating conditions. Whole-cell samples were prepared and subjected to GC/MS analysis as described in the Materials and Methods. (Asterisk), Methyl ester of PHB (methyl 3-hydroxybutanoate).

Sample Name : *M. smegmatis mc²155*
Injection Volume : 1.00

GC/MS Peak Report TIC

Peak#	R.Time	Area	Name	Area%
1	9.175	42433880	2-Furancarboxaldehyde	0.84
2	10.762	6901020	Ethylbenzene	0.14
3	11.243	32060657	Benzene, 1,2-dimethyl-	0.63
4	12.700	11532733	Benzene, 1,2-dimethyl-	0.23
5	16.497	13247578		0.26
6	16.641	5273001		0.10
7	17.641	244522219	Pentanoic acid, 4-oxo-, methyl ester	4.82
8	18.588	10751966	1-Hexanol, 2-ethyl-	0.21
9	18.676	56721540	Butanedioic acid, dimethyl ester	1.12
10	19.623	12399055		0.24
11	19.844	196172219	Decane, 2-mUndecane, C11ethyl-	3.87
12	20.198	4169067	Octanoic acid, methyl ester	0.08
13	20.626	5790970		0.11
14	20.789	8091122		0.16
15	21.582	27852754		0.55
16	21.927	21337654		0.42
17	22.078	23983693		0.47
18	22.281	23697288	Methyl 2,3,4-tri-O-methyl-6-deoxy-.alpha.-D-mannopyranoside	0.47
19	22.344	33698118		0.66
20	22.541	8563933	Decanoic acid, methyl ester	0.17
21	23.859	44577015	Dimethyl phthalate	0.88
22	24.263	5504149	9-Octadecenoic acid (Z)-, methyl ester	0.11
23	24.388	35344782	Dodecanoic acid, methyl ester	0.70
24	24.822	6439249	Dodecanoic acid, 4-methyl-, methyl ester	0.13
25	25.832	29065029	Eicosenoic acid, methyl ester	0.57
26	25.897	33876641	methyl myristoleate, C14:1 (cis-9) Me ester	0.67
27	26.026	254500211	Tridecanoic acid, 12-methyl-, methyl ester	5.02
28	26.239	5840412		0.12
29	26.348	24487564	Nonadecanoic acid, methyl ester	0.48
30	26.767	21543867	methyl pentadecanoate, C15:0 Me ester	0.42
31	27.025	5906772		0.12
32	27.416	673280705	Methyl palmitolenate, C16:1 (cis-9) Me ester	13.27
33	27.567	865089404	Pentadecanoic acid, 14-methyl-, methyl ester	17.06
34	27.789	247607764	Heptadecanoic acid, 10-methyl-, methyl ester	4.88
35	27.908	20612683		0.41
36	28.041	34116704	Cyclopropaneoctanoic acid, 2-hexyl-, methyl ester	0.67
37	28.180	32507892	Heptadecanoic acid, methyl ester	0.64
38	28.308	5579473		0.11
39	28.430	83852937		1.65
40	28.746	706768607	7-Octadecenoic acid, methyl ester	13.93
41	28.866	369671884	Heptadecanoic acid, 14-methyl-, methyl ester, (.+/-.)-	7.29
42	29.000	20587645		0.41
43	29.116	393934547	Octadecanoic acid, 10-methyl-, methyl ester	7.77
44	29.201	23939056	8-Heptadecene, 9-octyl-	0.47
45	29.666	103717879		2.04
46	29.933	4835622	11-Eicosenoic acid, methyl ester	0.10
47	30.072	87914539	methyl arachidate, C20:0 Me ester	1.73
48	30.142	5614221		0.11
49	31.213	44835265	Docosanoic acid, methyl ester	0.88
50	32.386	91191433	methyl lignocerate, C24:0 Me ester	1.80
		5071944418		100.00

FIG. S2. (*Continued*).

Sample Name : *M. smegmatis mc²155* (pMycVec2_P*wmyc_phaAB*) + pMV261_*phaC*
Injection Volume : 1.00

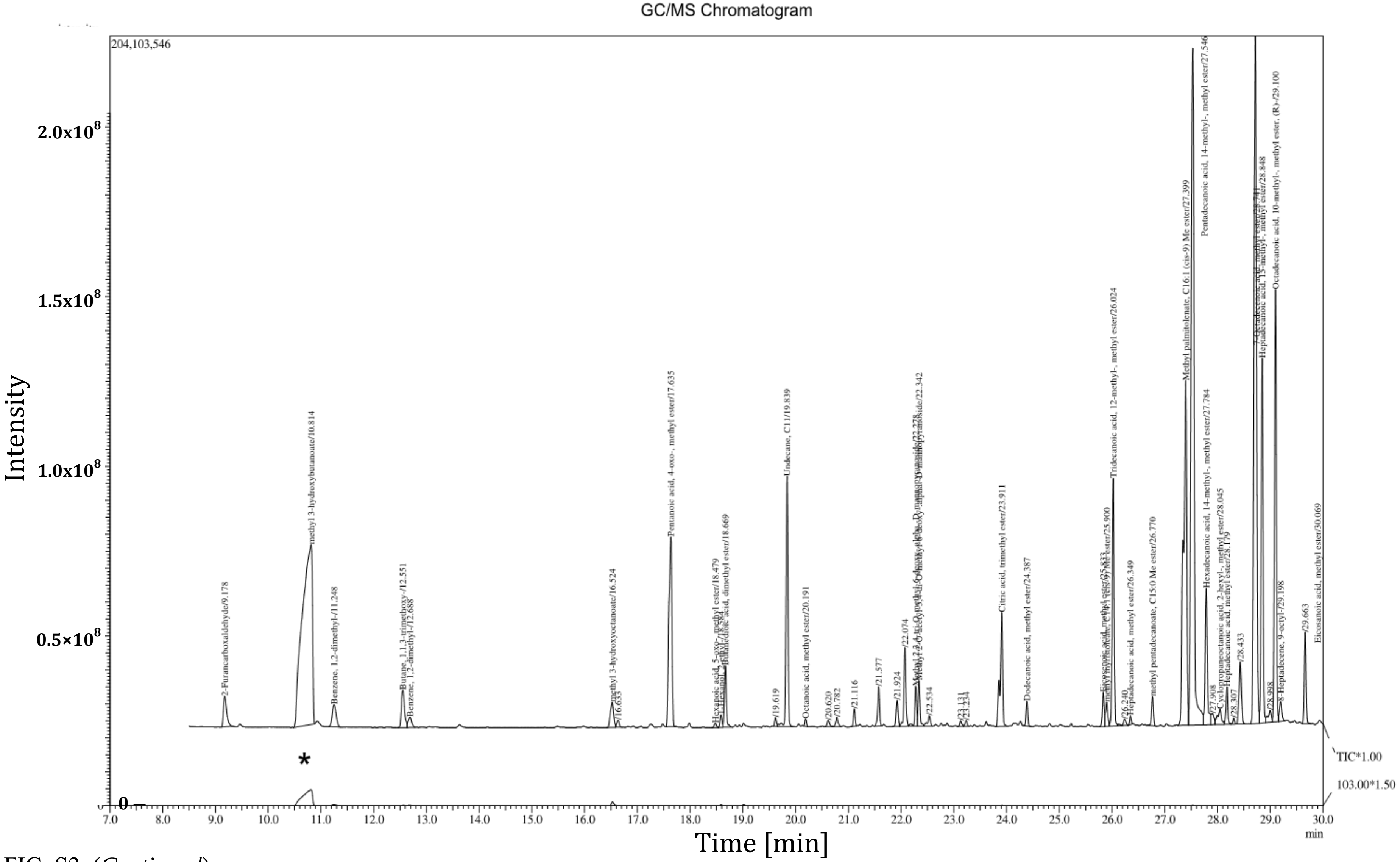

FIG. S2. (*Continued*).

Sample Name : *M. smegmatis mc²155* (pMycVec2_P*wmyc_phaAB*) + pMV261_*phaC*
Injection Volume : 1.00

GC/MS Peak Report TIC

Peak#	R.Time	Area	Name	Area%
* 2	10.814	708934395	methyl 3-hydroxybutanoate	14.75
1	9.178	38418036	2-Furancarboxaldehyde	0.80
3	11.248	33850792	Benzene, 1,2-dimethyl-	0.70
4	12.551	46522870	Butane, 1,1,3-trimethoxy-	0.97
5	12.688	13598686	Benzene, 1,2-dimethyl-	0.28
6	16.524	32550167	methyl 3-hydroxyoctanoate	0.68
7	16.633	6807288		0.14
8	17.635	233511530	Pentanoic acid, 4-oxo-, methyl ester	4.86
9	18.479	3647766	Hexanoic acid, 5-oxo-, methyl ester	0.08
10	18.584	9915279	1-Hexanol, 2-ethyl-	0.21
11	18.669	48132432	Butanedioic acid, dimethyl ester	1.00
12	19.619	6530655		0.14
13	19.839	200087099	Undecane, C11	4.16
14	20.191	4622523	Octanoic acid, methyl ester	0.10
15	20.620	6116816		0.13
16	20.782	8753277		0.18
17	21.116	11329132		0.24
18	21.577	29522286		0.61
19	21.924	20139395		0.42
20	22.074	60292183		1.25
21	22.278	29104957	Methyl 2,3,4-tri-O-methyl-6-deoxy-.alpha.-D-mannopyranoside	0.61
22	22.342	31668125	Methyl 2-O-acetyl-3,4-di-O-methyl-6-deoxy-.alpha.-D-mannopyranoside	0.66
23	22.534	11287773		0.23
24	23.131	4514535		0.09
25	23.234	4474010		0.09
26	23.911	115051862	Citric acid, trimethyl ester	2.39
27	24.387	16624556	Dodecanoic acid, methyl ester	0.35
28	25.833	25825525	Eicosenoic acid, methyl ester	0.54
29	25.900	20068668	methyl myristoleate, C14:1 (cis-9) Me ester	0.42
30	26.024	179221908	Tridecanoic acid, 12-methyl-, methyl ester	3.73
31	26.240	7283103		0.15
32	26.349	5586254	Heptadecanoic acid, methyl ester	0.12
33	26.770	21157027	methyl pentadecanoate, C15:0 Me ester	0.44
34	27.399	447883091	Methyl palmitolenate, C16:1 (cis-9) Me ester	9.32
35	27.546	678667701	Pentadecanoic acid, 14-methyl-, methyl ester	14.12
36	27.784	114506381	Hexadecanoic acid, 14-methyl-, methyl ester	2.38
37	27.908	12594137		0.26
38	28.045	23265343	Cyclopropaneoctanoic acid, 2-hexyl-, methyl ester	0.48
39	28.179	26837580	Heptadecanoic acid, methyl ester	0.56
40	28.307	4361956		0.09
41	28.433	58650185		1.22
42	28.741	574299558	7-Octadecenoic acid, methyl ester	11.95
43	28.848	275166774	Heptadecanoic acid, 15-methyl-, methyl ester	5.72
44	28.998	15177734		0.32
45	29.100	329042471	Octadecanoic acid, 10-methyl-, methyl ester, (R)-	6.85
46	29.198	17236494	8-Heptadecene, 9-octyl-	0.36
47	29.663	65392644		1.36
48	30.069	55866234	Eicosanoic acid, methyl ester	1.16
49	31.209	31257141	Docosanoic acid, methyl ester	0.65
50	32.375	81616650	methyl lignocerate, C24:0 Me ester	1.70
		4806972984		100.00

FIG. S2. (*Continued*).

Sample Name : *M. smegmatis mc²155* (pMycVec2_P*wmyc_phaAB*) + pMV261_A:E-*phaC*
Injection Volume : 1.00

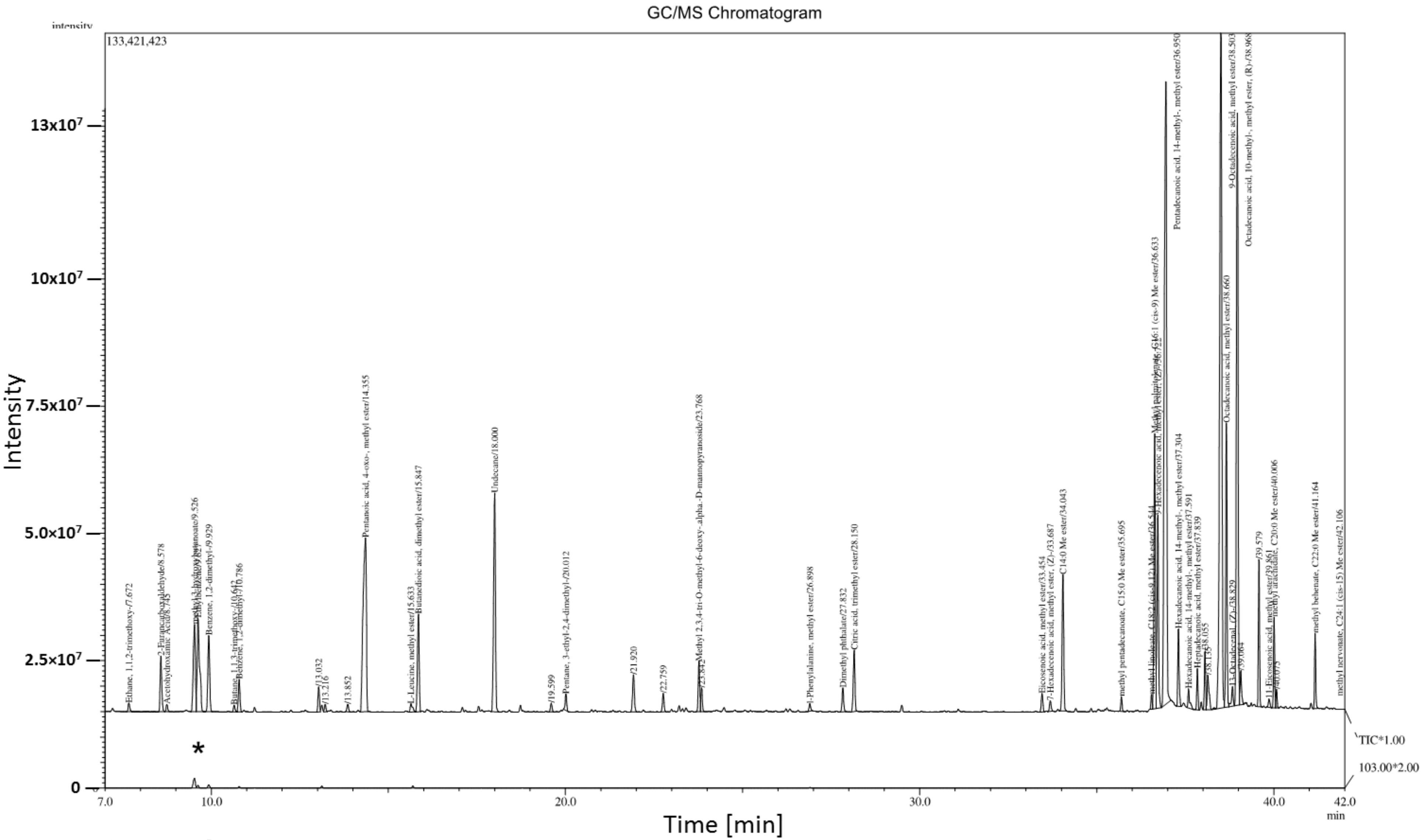

FIG. S2. (*Continued*).

Sample Name : *M. smegmatis mc²155* (pMycVec2_P*wmyc_phaAB*) + pMV261_A:E-*phaC*
Injection Volume : 1.00

GC/MS Peak Report TIC

	Peak#	R.Time	Area	Name	Area%
	1	4.199	3935885		0.12
	2	7.672	5269031	Ethane, 1,1,2-trimethoxy-	0.15
	3	8.578	33648244	2-Furancarboxaldehyde	0.99
	4	8.745	4252881	Acetohydroxamic Acid	0.12
*	5	9.526	71507176	methyl 3-hydroxybutanoate	2.10
	6	9.627	88003097	Ethylbenzene	2.58
	7	9.929	54604866	Benzene, 1,2-dimethyl-	1.60
	8	10.642	4236918	Butane, 1,1,3-trimethoxy-	0.12
	9	10.786	19821349	Benzene, 1,2-dimethyl-	0.58
	10	13.032	14607048		0.43
	11	13.216	4278135		0.13
	12	13.852	4733977		0.14
	13	14.355	218115592	Pentanoic acid, 4-oxo-, methyl ester	6.40
	14	15.633	7596528	L-Leucine, methyl ester	0.22
	15	15.847	64863201	Butanedioic acid, dimethyl ester	1.90
	16	18.000	157701476	Undecane	4.63
	17	19.599	5243040		0.15
	18	20.012	9543702	Pentane, 3-ethyl-2,4-dimethyl-	0.28
	19	21.920	25361132		0.74
	20	22.759	11275262		0.33
	21	23.768	29718255	Methyl 2,3,4-tri-O-methyl-6-deoxy-.alpha.-D-mannopyranoside	0.87
	22	23.842	12942496		0.38
	23	26.898	4628358	l-Phenylalanine, methyl ester	0.14
	24	27.832	14915984	Dimethyl phthalate	0.44
	25	28.150	40174392	Citric acid, trimethyl ester	1.18
	26	33.454	11140472	Eicosenoic acid, methyl ester	0.33
	27	33.687	7020500	7-Hexadecenoic acid, methyl ester, (Z)-	0.21
	28	34.043	86534504	C14:0 Me ester	2.54
	29	35.695	7440526	methyl pentadecanoate, C15:0 Me ester	0.22
	30	36.544	7075596	methyl linoleate, C18:2 (cis-9,12) Me ester	0.21
	31	36.633	151999448	Methyl palmitolenate, C16:1 (cis-9) Me ester	4.46
	32	36.722	113611806	9-Hexadecenoic acid, methyl ester, (Z)-	3.33
	33	36.950	593128248	Pentadecanoic acid, 14-methyl-, methyl ester	17.41
	34	37.304	40246214	Hexadecanoic acid, 14-methyl-, methyl ester	1.18
	35	37.591	12569515	Hexadecanoic acid, 14-methyl-, methyl ester	0.37
	36	37.839	18709285	Heptadecanoic acid, methyl ester	0.55
	37	38.055	28178130		0.83
	38	38.135	24801999		0.73
	39	38.503	593781453	9-Octadecenoic acid, methyl ester	17.43
	40	38.660	142049873	Octadecanoic acid, methyl ester	4.17
	41	38.829	13382966	13-Octadecenal, (Z)-	0.39
	42	38.968	400657246	Octadecanoic acid, 10-methyl-, methyl ester, (R)-	11.76
	43	39.064	20162647		0.59
	44	39.579	69190080		2.03
	45	39.861	5137947	11-Eicosenoic acid, methyl ester	0.15
	46	40.006	42542562	methyl arachidate, C20:0 Me ester	1.25
	47	40.075	7834610		0.23
	48	41.164	35132887	methyl behenate, C22:0 Me ester	1.03
	49	42.106	6741763	methyl nervonate, C24:1 (cis-15) Me ester	0.20
	50	42.215	56997562	methyl lignocerate, C24:0 Me ester	1.67
			3407045864		100.00

FIG. S2. (*Continued*).

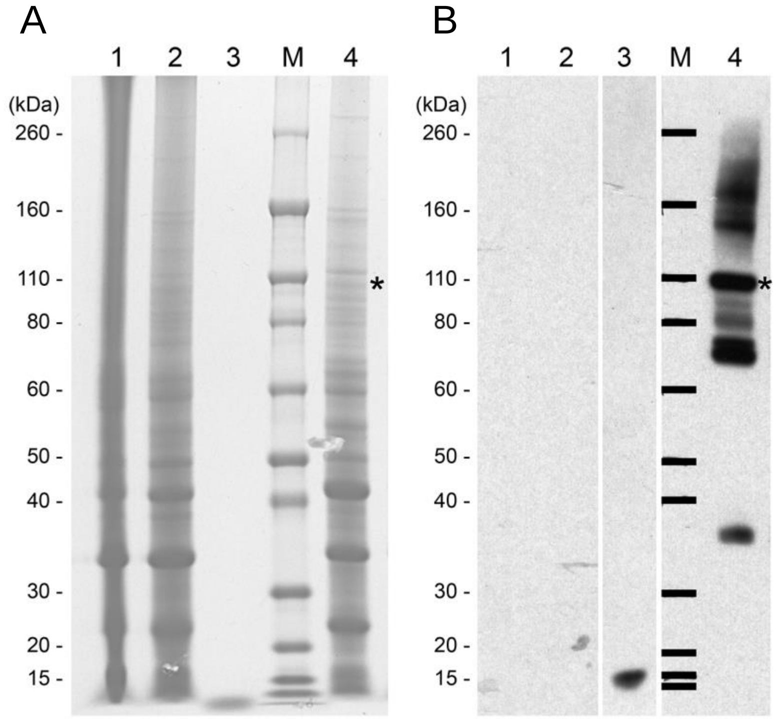

FIG. S3. Confirmation of ESAT-6. *M. smegmatis* harboring various plasmid systems regulated under nitrile inducible promoter (pNit) were grown under protein inducive conditions. (A) SDS-PAGE and (B) immunoblot with anti-ESAT-6 polyclonal Ab. Samples were arranged accordingly: lane 1, pMycVec2_P*wmyc-phaAB* and pMV261_*phaC* (MBB), lane 2, pMycVec2_P*wmyc-phaAB* (MVC) negative control; lane 3, ESAT-6 postive control; lane M, molecular weight standard; lane 4, pMycVec2_P*wmyc-phaAB* and pMV261_A:E-*phaC* (A:E-MBB). Asterisk indicates A:E-PhaC protein.

Sample Name : *E. coli* BL21 (pMCS69) + pMIND_pTet-*phaC*
Injection volume : 1.00

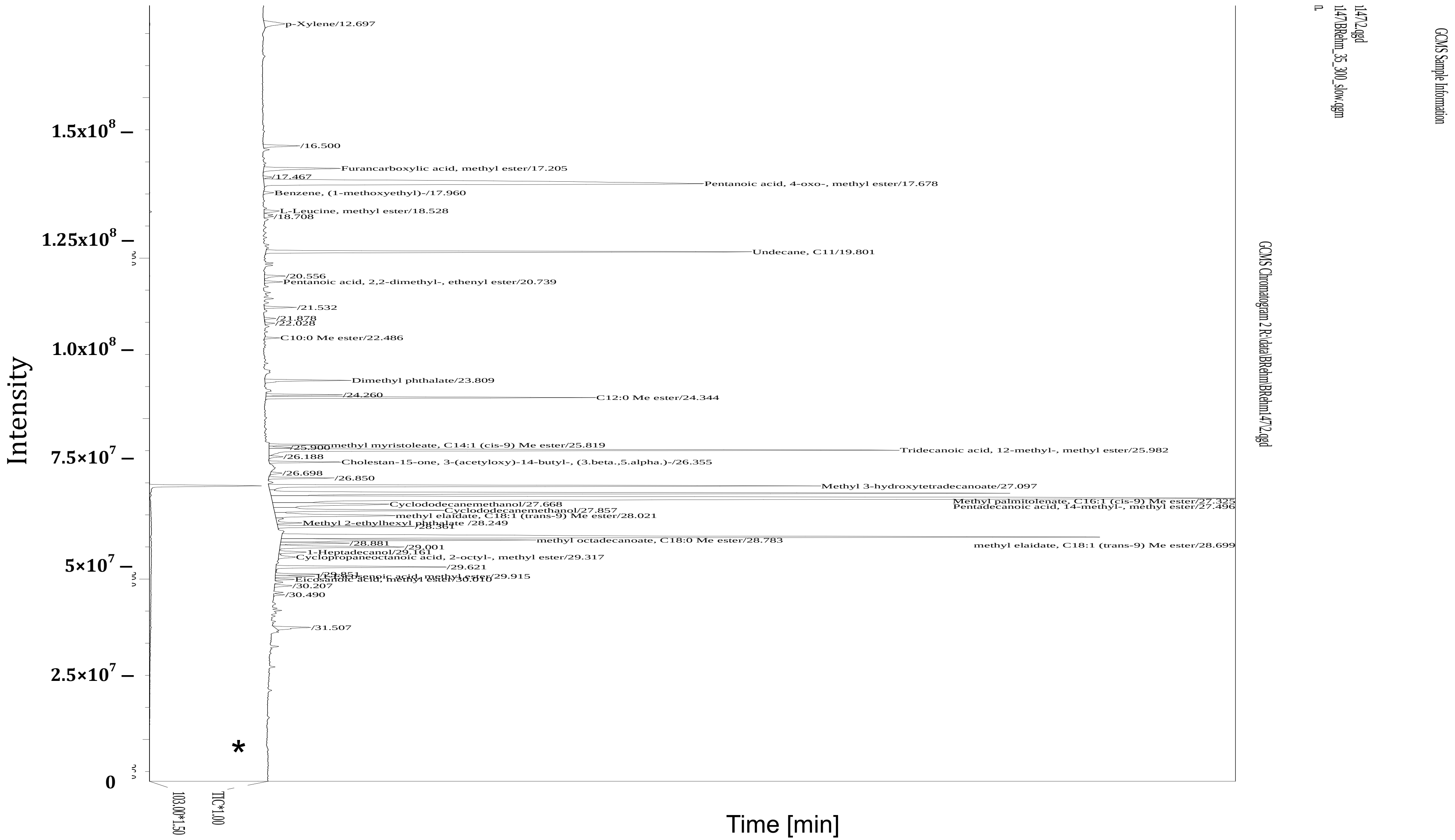

FIG. S4. Analysis of PHB in whole-cell by GC/MS. *E. coli* BL21 harboring plasmids pMCS69 and pMIND_pTet-*phaC* were cultivated under PHB accumulating conditions. Whole-cell samples were prepared and subjected to GC/MS analysis as described in the Materials and Methods. (Asterisk), Methyl ester of PHB (methyl 3-hydroxybutanoate).

Sample Name : *E. coli* BL21 (pMCS69) + pMIND_pTet-*phaC*
Injection volume : 1.00

GCMS Sample Information

Sample Name : 2

Injection Volume : 1.00
Acquisition Date : 28/07/2013 2:32:04 p.m.
Vial # : 2
Data File : R:\data\BRehm\BRehm147\2.qgd
Method File : R:\data\BRehm\BRehm147\BRehm_35_300_slow.qgm
Report File : R:\data\BRehm\BRehm147\landscape, lib search report & specified ion.qgr

GCMS Peak Report TIC

Peak#	R.Time	Area	Name	Area%
1	9.330	17839811	2-Furancarboxaldehyde	0.41
2	10.784	141640623	methyl 3-hydroxybutanoate	3.23
3	11.027	69129741	Sulfuric acid, dimethyl ester	1.58
4	11.280	54579662	Benzene, 1,2-dimethyl-	1.24
5	12.697	21930778	p-Xylene	0.50
[illegible]	[illegible]	18041122		0.41
		44269514	Furancarboxylic acid, methyl ester	1.01
		4400270		0.10
		483386702	Pentanoic acid, 4-oxo-, methyl ester	11.02
		4886808	Benzene, (1-methoxyethyl)-	0.11
		9203406	L-Leucine, methyl ester	0.21
		7594987		0.17
		218217837	Undecane, C11	4.98
		10587835		0.24
		8017009	Pentanoic acid, 2,2-dimethyl-, ethenyl ester	0.18
		12750456		0.29
		4439990		0.10
		4096429		0.09
		5554627	C10:0 Me ester	0.13
		35613445	Dimethyl phthalate	0.81
		28515207		0.65
		127257642	C12:0 Me ester	2.90
		22075866	methyl myristoleate, C14:1 (cis-9) Me ester	0.50
		9627947		0.22
		287107573	Tridecanoic acid, 12-methyl-, methyl ester	6.55
		6496418		0.15
		26543896	Cholestan-15-one, 3-(acetyloxy)-14-butyl-, (3.beta.,5.alpha.)-	0.61
		5231724		0.12
		23992020		0.55
		242222700	Methyl 3-hydroxytetradecanoate	5.52
		373885099	Methyl palmitolenate, C16:1 (cis-9) Me ester	8.53
		835402100	Pentadecanoic acid, 14-methyl-, methyl ester	19.05
		90868584	Cyclododecanemethanol	2.07
		101056175	Cyclododecanemethanol	2.30
		81495977	methyl elaidate, C18:1 (trans-9) Me ester	1.86
		13943365	Methyl 2-ethylhexyl phthalate	0.32
		58026807		1.32
		497960844	methyl elaidate, C18:1 (trans-9) Me ester	11.36
		114311012	methyl octadecanoate, C18:0 Me ester	2.61
		29346647		0.67
		62460134		1.42
		14385251	1-Heptadecanol	0.33
		10511719	Cyclopropaneoctanoic acid, 2-octyl-, methyl ester	0.24
		73045028		1.67
		17748660		0.40
		14411642	11-Eicosenoic acid, methyl ester	0.33
		7005998	Eicosanoic acid, methyl ester	0.16
		9040735		0.21
		3811359		0.09
		21285235		0.49
		[illegible]385254416		100.00

*

FIG. S4. (*Continued*).
